# Effectiveness of Treatment in Children With Cerebral Palsy

**DOI:** 10.7759/cureus.13754

**Published:** 2021-03-07

**Authors:** Syed Faraz Ul Hassan Shah Gillani, Akkad Rafique, Muhammad Taqi, Muhammad Ayaz ul Haq Chatta, Faisal Masood, Tauseef Ahmad Blouch, Syed Muhammad Awais

**Affiliations:** 1 Orthopedic Surgery, King Edward Medical University/Mayo Hospital Lahore, Lahore, PAK; 2 Orthopedics and Traumatology, Mohterma Benazir Bhutto Medical Shaheed College, Mirpur Azad Jammu Kashmir, PAK; 3 Orthopedics and Traumatology, King Edward Medical University/Mayo Hospital Lahore, Lahore, PAK; 4 Surgery, King Edward Medical University/Mayo Hospital Lahore, Lahore, PAK; 5 Orthopaedic Surgery, King Edward Medical University/Mayo Hospital Lahore, Lahore, PAK

**Keywords:** cerebral palsy, congenital disease, central nervous system, motor impairment syndrome, gait analysis

## Abstract

Objective: The objective of this study was to assess the effectiveness of conservative and surgical treatment in cerebral palsy children by evaluating the Medical Research Council (MRC) grading system, modified Ashworth scale, and Barthel Activities of Daily Life (ADL) scale.

Method: This prospective case series was performed using a non-probability consecutive sampling technique at the Department of Orthopedic Surgery and Traumatology, King Edward Medical University/Mayo Hospital, Lahore from October 2011 to November 2013. Two hundred children of all ages, having cerebral palsy diagnosed on history and clinical examination were enrolled in the study. Children were treated with conservative and surgical treatment. Pre- and post-treatment, all children were classified based on movement disorder (spastic, athetoid, ataxic, and mixed), parts of the body involved (paraplegic, tetraplegic, diplegic, hemiplegic, monoplegic, double hemiplegic, and triplegic), and gross motor function (GMFCS level I-IV). Their muscle power and tone were assessed using the MRC grading system and modified Ashworth scale, respectively. Assessment of disability and daily function was done by ranking disability grading and Barthel ADL, respectively. The range of motion (ROM) of each joint was assessed clinically. Children were divided based on the treatment method as non-surgical versus surgical treatment.

Results: Out of a total of 200 children, the mean age of the children was 7.86±4.17year. There were 134 (67.0%) males and 66 (33.0%) female children. Classification on basis of movement disorder, body part involved, and gross motor function at three-month intervals till twelve months was performed. From the first presentation of children till the last follow-up time period, i.e., 12th month there was no change in the movement disorder (a type of CP, body parts involved, and GMFCS). The final rating of overall treatment results shows that there were 84 (42%) patients who had a poor outcome, and only 35 (17.50%) patients had a fair treatment outcome and 81 (40.50%) patients had good treatment outcomes.

Conclusion: The conservative and surgical management showed no effect on movement disorder of the child although, on the final rating scale fair to good treatment outcome was observed in all children. There was an improvement in muscle power grading on the ADL, but no significant improvement was seen on the improvement of type, parts of the body involved, gross motor function classification, modified Ashworth, and ranking disability grading of the children.

## Introduction

Cerebral palsy (CP) is a group of non-progressive disorders of the brain and nervous system dysfunction. It affects the child's movement, vision, learning, and thinking processes [1]. It can occur before birth, during birth, and within the first year of childbirth [2]. It appears as a result of brain injuries and abnormalities that can present two years after birth. In some children, it occurs as a result of brain hypoxia while premature infants have a higher risk of developing CP [3,4].

The reported incidence of CP is 1.5 to 2.5 per 1000 childbirth. These incidences were higher between 1960 and 1980. Now, data have reported 0.5 per 1000 childbirths [5]. Diplegic CP has a 95% survival rate versus 75% in quadriplegic children till the age of 30 years. Survival of mild to severe mental retard rate has been reported as 65% and 90%, respectively, till the age of 38 years with an overall survival rate is 90% till the age of 20 years [6].

CP is the second most common disease after mental retardation that causes disability in child growth, but it also affects adults. There is a lack of special training amongst primary healthcare physicians and specialists for treating children with developmental abnormalities [7]. The symptoms of CP vary according to the presentation. It can be mild to severe with one side or whole of the body involvement [8]. Increasing research work to understand the hormonal, infection, and genetic factors are involved in premature births of babies given by the mother [9,10].

The goal for these children, their families, medical care, education, and society at large is for them to grow and develop to their maximum capabilities so that they may succeed as contributing members of society. We studied the conservative and surgical treatment outcome of CP children. It helped us understand the problem of the CP, treatment options, and their outcomes with the passage of time. 

## Materials and methods

This prospective case series was done using a non-probability consecutive sampling technique at the Department of Orthopedic Surgery and Traumatology, King Edward Medical University/Mayo Hospital, Lahore from October 2011 to November 2013. Two hundred children of all ages, having CP diagnosed on history and clinical examination were enrolled in the study. Children with complications (bed sores), systemic illness (diabetes mellitus, ischemic heart disease, asthma, Hepatitis B, and Hepatitis C) skin disease, and malignancy were excluded from the study.

After obtaining ethical permission from the university institutional review board (IRB), informed written consent was obtained from parents of the children. We obtained demographic data of all the children. Pre-treatment, all children were classified based on movement disorder (spastic, athetoid ataxic, and mixed), parts of the body involved (paraplegic, tetraplegic, diplegic, hemiplegic, monoplegic, paraplegic, double hemiplegic, and triplegic), and gross motor function (GMFCS level I-IV). Their muscle power and tone were assessed using the Medical Research Council (MRC) grading system and modified Ashworth scale, respectively. Assessment of disability and daily function was done by ranking disability grading and Barthel Activities of Daily Life (ADL), respectively. The range of motion (ROM) of each joint was assessed clinically. Children were treated with conservative and surgical treatment methods.

By non-surgical treatment we meant, rehabilitation, physical therapy, speech therapy, occupational therapy, medical, splinting, casting, braces or orthosis, and other treatments. By surgical treatment, we included tenotomy, tendon lengthening, tendon transfer, muscle lengthening, neurectomy, capsulotomies, osteotomies, arthrodesis, contracture release, selective rhizotomy, multi-level surgery, and spine surgery.

Children were followed up in the out-patient department at a three-month interval for twelve months. They were evaluated based on movement disorder (spastic, athetoid ataxic, and mixed), body part involved (paraplegic, tetraplegic, diplegic, hemiplegic, monoplegic, paraplegic, double hemiplegic, and triplegic), GMFCS level I-V, muscle power by MRC grading, tone by modified Ashworth score. Disability and activity of daily life were assessed using ranking disability grading and Barthel ADL chart, respectively. Active and passive ROM, gait patterns, and complications were recorded clinically.

Data obtained were coded and tabulated on the spreadsheet. SPSS version 18 (IBM Corp., Armonk, NY) was used to analyze data (continuous variable age, height, and weight) that were presented in the form of a table and histogram. Mean, mode, range, and stranded deviation were used to see the central tendency and measures of dispersion. The descriptive statistics of the categorical variable was present in the form of ratio, graphs, and tables.

## Results

Children's routine investigations were done. Routine investigations include a comparison of the type of non-surgical and surgical treatments used for children with CP which was enlisted in Table 1. The type of CP, type of body part involved, gross motor function classification, and special tests of children with CP before treatment are given in Table 2.

**Table 1:** Non-surgical and surgical treatment of cerebral palsy children

Variables	Yes	No	Total
Non-surgical treatment rehabilitation	78	122	200
Physical therapy	182	18	200
Occupational therapy	42	158	200
Speech therapy	62	138	200
Medical therapy	172	28	200
Splints	35	165	200
Casts	24	176	200
Braces or orthosis	47	153	200
Other	00	200	200
Tenotomy	15	185	200
Tendon lengthening	46	154	200
Tendon transfer	10	190	200
Surgical treatment muscle lengthening	42	158	200
Neurectomy	20	180	200
Capsulotomies	07	193	200
Osteotomies	19	181	200
Arthrodesis	2	198	200
Selective rhizotomy	00	200	200
Multilevel surgery	00	200	200
Spine surgery	00	200	200

**Table 2:** Pre-treatment classification based on type, the involvement of body parts, and gross motor function and special test in children with cerebral palsy.

Variables	Frequency	Percentage (%)
Types of cerebral palsy		
Spastic	166	83%
Athetoid	27	13.5%
Ataxic	04	02%
Mixed	03	02%
Involvement of body part		
Triplegic	11	5.5%
Paraplegic	73	36.5%
Tetraplegic	35	17.5%
Diplegic	20	10%
Hemiplegic	13	6.5%
Paraplegic	20	10%
Monoplegic	24	12%
Double hemiplegic	04	02%
Gross Motor Function Classification System		
Level-1	15	7.5%
Level-2	30	15%
Level-3	45	22.5%
Level-4	55	27.5%
Level-5	55	27.5%
Special test		
Magnetic resonance image	05	1.75%
Computed tomography	51	17.9%
Electroencephalogram	12	4.2%
Electromyography	36	12.6%
Ultrasonogram	36	12.6%
Audiometry	30	10.5%
Speech test	42	14.8%
Radiographs	73	25.6%

On follow-up, classification on basis of movement disorder, body part involved, and gross motor function at three-month intervals till twelve months was done. From the first presentation of children till the last follow-up time period, i.e., 12th month, there was no change in the movement disorder (Table 3).

**Table 3:** Classification on basis of movement disorder, body parts involvement, and gross motor function improvement at different time intervals on the follow-up of children.

	Presentation	3^rd^ month	6^th ^month	9^th^ month	12^th^ month
Movement disorders					
Spastic	162	162	162	162	162
Athetoid	31	31	31	31	31
Ataixic	04	04	04	04	04
Mixed	03	03	03	03	03
Body parts involvement					
Monoplegia	20	20	20	20	20
Hemiplegia	13	13	13	12	12
Paraplegia	73	73	73	73	73
Diplegia	20	20	20	20	20
Triplegia	11	11	11	10	10
Quadriplegia	35	35	35	30	30
Paraplegia	24	24	24	20	16
Double hemiplegia	04	04	04	04	04
Gross motor function					
Level-I	15	15	15	15	15
Level-II	30	30	27	25	22
Level-III	45	45	40	38	35
Level-IV	55	55	45	40	40
Level-V	55	55	47	42	35

The ROM was assessed for 12 months follow-up time period. In the 3rd month, there were 92 children whose ROM was increased, at 6th month, there were 177 children whose ROM was increased, at 9th month, there were 154 children whose ROM was increased, and in the 12th month, there was a total of 181 children whose ROM was increased. It shows that after treatment ROM improves with the passage of the follow-up time period.

Assessment of muscle power grading, muscle tone, disability, and activity of daily life was done using the MRC scale, modified Ashworth scale, ranking disability grading, and Barthel ADL, respectively. Scoring was done on the first presentation until the last follow-up. The effects of treatment have been given in Table 4.

**Table 4:** Pre-operative and post-operative functional scoring system

Variables	Pre-operative	3^rd^ month	6^th^ month	9^th^ Month	12^th^ Month
Medical Research Council scale	2.94±0.76	2.81±0.41	2.83±0.43	2.83±0.43	3.52±0.53
Modified Ashworth scale	2.81±0.41	2.94±0.76	2.93±0.76	2.91±0.77	2.72±0.85
Ranking disability grading	3.75±1.05	3.73±1.04	3.60±1.03	3.40±1.130	3.12±1.12
Barthel activities of daily life	7.33±6.65	7.62±6.63	8.77±6.94	9.81±7.09	10.70±7.05

The gait of children at different time intervals during the follow-up time period was observed. Limping gait was improved from 41 children at 3rd months to 29 children at 12th months of follow-up. Dancing gait was present in 13 children and ataxic gait in 7 children until the last follow-up time period. There were 15 children in which crouch gait was observed in the 3rd month and at the 12th-month crouch gait was observed in 9 children. At 3rd and 6th month, 87 children were bedridden, at 9th month 80 children and at 12th month 73 children were bedridden. Similarly, 37 children do crawl at 3rd month and till the 12th months, 28 Children do crawl.

In the 3rd month, 13 children had complications, at the 6th and 9th month, 8 children had complications, and at the 12th month, only 7 Children had complications. The final rating of overall treatment results shows that there were 84 (42%) children who had poor outcomes, only 35 (17.50%) Children had a fair treatment outcome, and 81 (40.50%) Children had good treatment outcomes.

## Discussion

In every 1000 live births, the CP rate as a whole was between 2 and 3. But may increase up to 40-100 per 1000 live births in premature and low birth weight newborns [11]. Diplegia, hemiplegia, and quadriplegia (involvement of all four limbs) were involved to explain the clinical patterns of CP. The term double hemiplegia suggested bilateral involvement described by greater involvement of the arms than the legs. Asymmetrical involvement or a clearly dominant side was suggested by some practitioners to explain this term. Spasticity, rigidity, hypotonia, dystonia (e.g., athetosis), or a mixture of these disorders were some movement disorders and they can coexist with clinical patterns [12].

Lack of specific diagnosis procedures for diplegia, quadriplegia, and hemiplegia was very difficult to access for clinical processing. The distribution of clinical patterns was identified in a study in France done by Bleck and the percentage that came out was quadriplegia was 40%, diplegia was 17%, and hemiplegia was present in 21% of children [13]. The Central West Health Planning Information Network (Ontario, Canada) reported that the percentage of non-specific classified children was 45·7%. The Canchild Centre for Childhood Disability in Ontario reported the gross motor function classification system; they took 408 children for this purpose and divided into five different levels: level I, 112 (27·5%); level II, 47 (11·5%); level III, 81 (19·9%); level IV, 82 (20·1%); and level V, 86 (21·1%). Level I indicates few limitations and level V severe impairments, respectively [14].

Compared to the findings of our study classification 20(10%) children had monoplegia, 13 (6.50%) children were hemiplegic, 73(36.50%) were paraplegic, 20(10%) were diplegic, 11(5.50%) were triplegic, 35(17.50%) children were quadriplegic. According to the valuation of 906 children in central and western North Carolina, USA, 294 (33%) had diplegia, 210 (23%) hemiplegia, and 402 (44%) quadriplegia [15].

Clinical findings were used primarily for the determination of CP as the spasticity management technique in children. Spasticity, muscle stiffness, muscle contracture, dynamic and static joint deformity, torsional deformities, and abnormal motor control are some of the parameters which were mandatory for the treatment of musculoskeletal abnormalities. The use of a continuum of modalities throughout childhood was one of the basic management for spasticity; a combination of modalities was seen to be used in the majority of children. It was investigated that small doses of drugs given orally for reduction of spasticity were effective comparatively. It was concluded that the botulinum toxin injection was used intramuscularly for the management of focal spasticity. The effect of the injection could be increased if it was associated with physiotherapy and orthotics as well [16-18].

Occupational therapy, physiotherapy, and speech therapy, device-assisted modalities (e.g., electrical stimulation), orthotics, casting, or any combination of these methods are the non-pharmacological approaches used for treatment. Joint ROM, facilitate or strengthen weak muscles, inhibit or weaken spastic agonist muscles, provide support, improve muscle strength, and improve or normalize motor development.

The surgical methods, which were used, for the treatment of children, included tenotomy, tendon lengthening, tendon transfer, muscle lengthening, neurectomy, capsulotomies, osteotomies, and arthrodesis. Significant improvement was seen as a result of these treatment modalities. Muscle power, muscle tone, ROM, and gait analysis were improved.

Physiotherapy, occupational, and/or speech were given to most of the children suffering from CP. There is little evidence-based information supporting or refuting their usage in the management of CP [19-21]. Maximum benefits were attained from physiotherapy and occupational therapy that were important factors for successful medical and surgical intervention [22]. Muscle-strengthening has been associated with improved function in children with CP [23]. Strength training has been advocated to promote fitness and increase children’s participation in various recreational and occupational activities. Physiotherapy and occupational therapy interventions include a wide range of techniques and schools including classic neurodevelopmental therapy techniques [24,25] and constraint-induced movement [26].

Constraint-induced therapy treatment models developed for rehabilitation of the arm in adults with stroke are now under investigation for use in children with CP. Preliminary case studies or trials with small numbers of children suggest that constraint-induced therapy results in improvements in hand function. However, further research is needed to define the long-term effects of this intervention in children with CP [27].

Our data have not shown the good effect of treatment on movement disorder of the CP children although there was an improvement in muscle power and improvement on the activity of daily life of CP in our study. Our study has a short follow-up to observe the treatment outcome of CP. Large follow can better predict the effect of conservative and surgical treatment outcomes in CP children.

## Conclusions

Both treatments (conservative and surgical) have a good effect on the final rating scale of treatment on CP child ranging from fair to good outcomes. There was an improvement in muscle power grading on the ADL, but no significant improvement was seen on the improvement of type, parts of the body involved, gross motor function classification, modified Ashworth, and ranking disability grading of the children.

## References

[1] Johnston MV (2011). Encephalopathies. Nelson Textbook of Pediatrics.

[2] Evans PM, Evans SJ, Alberman E (1990). Cerebral palsy why we must plan for survival. Arch Dis Child.

[3] Murphy CC, Yeargin-Allsopp M, Decouflé P, Drews CD (1993). Prevalence of cerebral palsy among ten-year-old children in metropolitan Atlanta, 1985 through 1987. J Pediatr.

[4] Parker GM, Hrist M (1997). Continuity and changes in medical care for young adult with disabilities. J Royal Coll Physicians Lond.

[5] Escobar GJ, Littenberg B, Petitti DB (1991). Outcome among surviving very low birthweight infants: a meta-analysis. Arch Dis Child.

[6] Stanly FJ (1992). Survival and cerebral palsy low birth infants: implication of perianatal care. Paediatr Perinat Epidemiol.

[7] Parker S, Greer S, Zuckerman B (1998). Double jeopardy: the impact of poverty on early child development. Pediatr Clin North Am.

[8] Ashwal S, Russman BS, Blasco PA (2004). Practice parameter: diagnostic assessment of the child with cerebral palsy: report of the Quality Standards Subcommittee of the American Academy of Neurology and the Practice Committee of the Child Neurology Society. Neurology.

[9] Scher AI, Petterson B, Blair E (2002). The risk of mortality or cerebral palsy in twins: a collaborative population-based study. Perdiatr Res.

[10] Nelson KB, Ellenberg JH (1978). Epidemiology of cerebral palsy. Adv Neurol.

[11] Surveillance of Cerebral Palsy in Europe (2007). Surveillance of cerebral palsy in Europe: a collaboration of cerebral palsy surveys and registers. Surveillance of Cerebral Palsy in Europe (SCPE). Dev Med Child Neurol.

[12] Dias RC, Miller F, Dabney K, Lipton G, Temple T (1996). Surgical correction of spinal deformity using a unit rod in children with cerebral palsy. J Pediatr Orthop.

[13] Bleck EE (1979). Orthopaedic management of cerebral palsy (Saunders monographs in clinical orthopaedics; v.2). https://www.amazon.com/Orthopaedic-management-cerebral-monographs-orthopaedics/dp/0721617301..

[14] Hagberg B, Hagberg G, Olow I (1993). The changing panorama of cerebral palsy in Sweden. VI. Prevalence and origin during the birth year period 1983-1986. Acta Paediatr.

[15] Pharoah PO, Cooke T, Cooke RW, Rosenbloom L (1990). Birthweight specific trends in cerebral palsy. Arch Dis Child.

[16] Rumeau-Rouquette C, Grandjean H, Cans C, Mazaubrun C, Verrier A (1997). Prevalence and time trends of disabilities in school-age children. Int J Epidemiol.

[17] Goldstein EM (2001). Spasticity management: an overview. J Child Neurol.

[18] O'Brien CF (2002). Treatment of spasticity with botulinum toxin. Clin J Pain.

[19] Graham HK, Aoki KR, Autti-Rämö I (2000). Recommendations for the use of botulinum toxin type A in the management of cerebral palsy. Gait Posture.

[20] Bower E, Michell D, Burnett M, Campbell MJ, McLellan DL. (2001). Randomized controlled trial of physiotherapy in 56 children with cerebral palsy followed for 18 months. Dev Med Child Neurol.

[21] Butler C, Darrah J (2001). Effects of neurodevelopmental treatment (NDT) for cerebral palsy: an AACPDM evidence report. Dev Med Child Neurol.

[22] Palmer FB, Shapiro BK, Wachtel RC (1988). The effects of physical therapy on cerebral palsy. A controlled trial in infants with spastic diplegia. N Engl J Med.

[23] Damiano DL, Dodd K, Taylor NF (2002). Should we be testing and training muscle strength in cerebral palsy?. Dev Med Child Neurol.

[24] Bobath B (1969). The treatment of neuromuscular disorders by improving patterns of co-ordination. Physiotherapy.

[25] Knox V, Evans AL (2002). Evaluation of the functional effects of a course of Bobath therapy in children with cerebral palsy: a preliminary study. Dev Med Child Neurol.

[26] Charles J, Lavinder G, Gordon AM (2001). Effects of constraint-induced therapy on hand function in children with hemiplegic cerebral palsy. Pediatric Physical Therapy.

[27] Huang H, Fetters L, Hale J (2009). Bound for success: a systematic review of constraint-induced movement therapy in children with cerebral palsy supports improved arm and hand use. Phys Ther.

